# Supplementary material for: Shoulder Positioning during Superior Capsular Reconstruction: Computational Analysis of Graft Integrity and Shoulder Stability
Source: Biology (Basel). 2021 Dec 3;10(12):1263. doi: 10.3390/biology10121263 (PMC8698921; doi:10.3390/biology10121263)
Supplement: Supplementary file 1 [file biology-10-01263-s001.zip › biology-1456039-supplementary.pdf]

Shoulder positioning during superior capsular reconstruction: computational analysis of graft integrity and shoulder stability

Supplementary materials

3. Results

3.1. Risk of tear

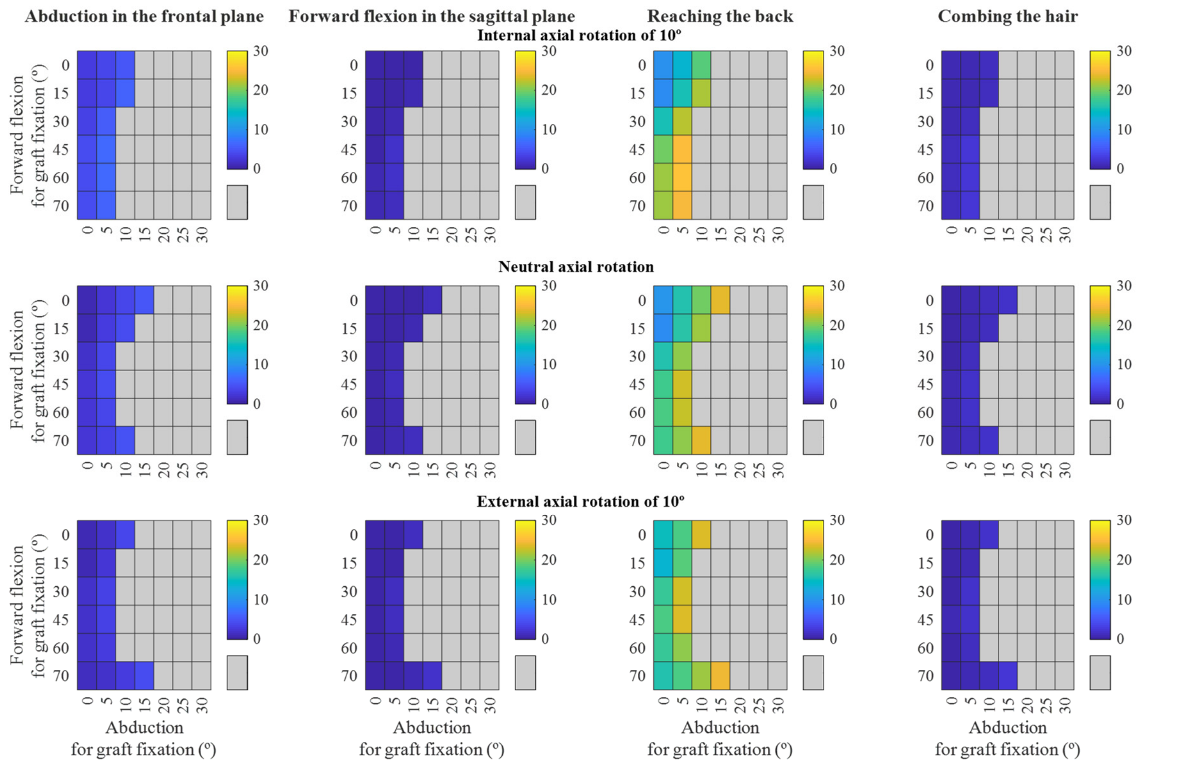

(a)

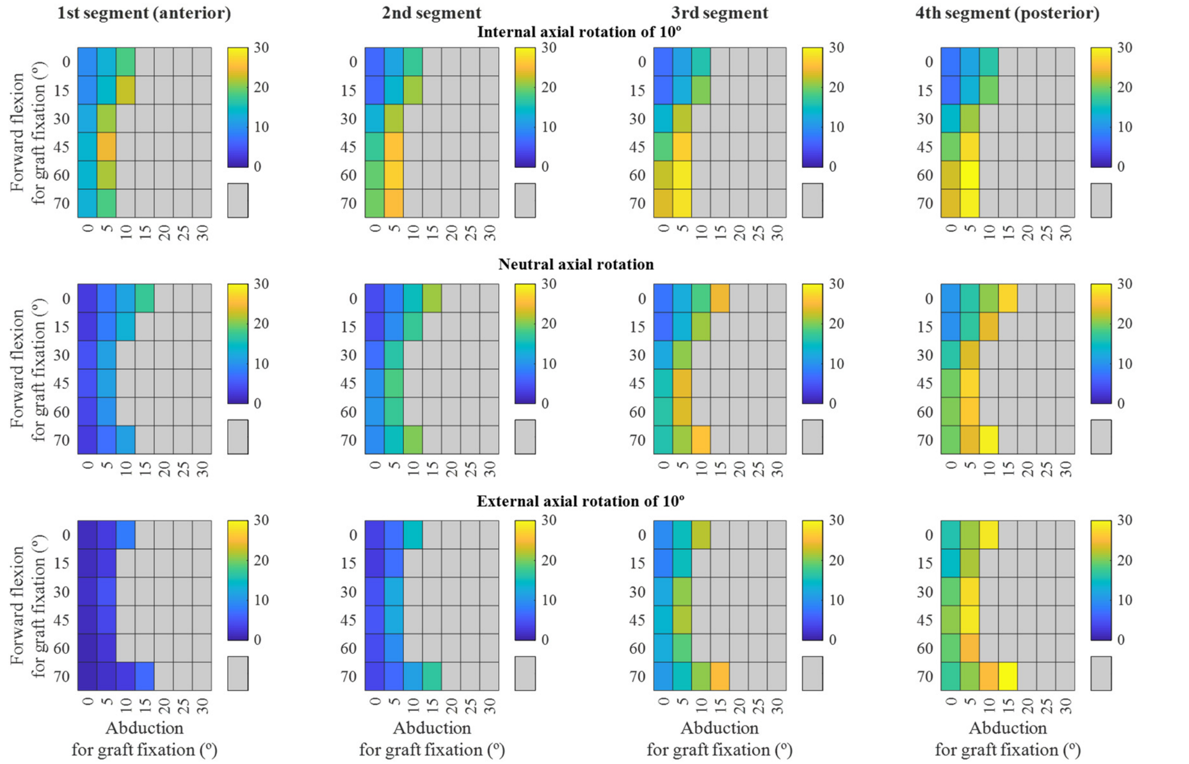

(b)

Figure S1. Incidence of the high risk of tear motion instants (%), for each shoulder position of fixation, between the (a) four studied movements and (b) four segments of the graft. The normalization factor used was 7519, the total amount of evaluated motion instants. The grey color represents the shoulder positions of fixation that failed the screening step.

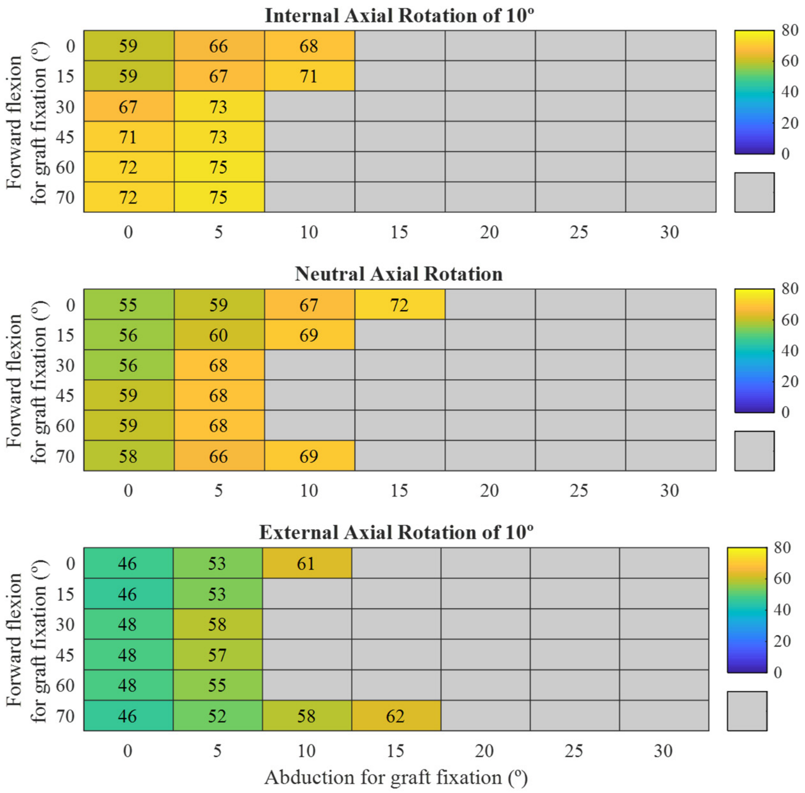

Figure S2. Average amount of segments at risk of tear (%), for each shoulder position of fixation. The normalization factor for each shoulder position of fixation corresponds to the respective number of high risk of tear motion instants, for which the strain of at least one segment was above the limit of failure. The grey color represents the shoulder positions of fixation that failed the screening step.

3.2. Biomechanical performance of ASCR

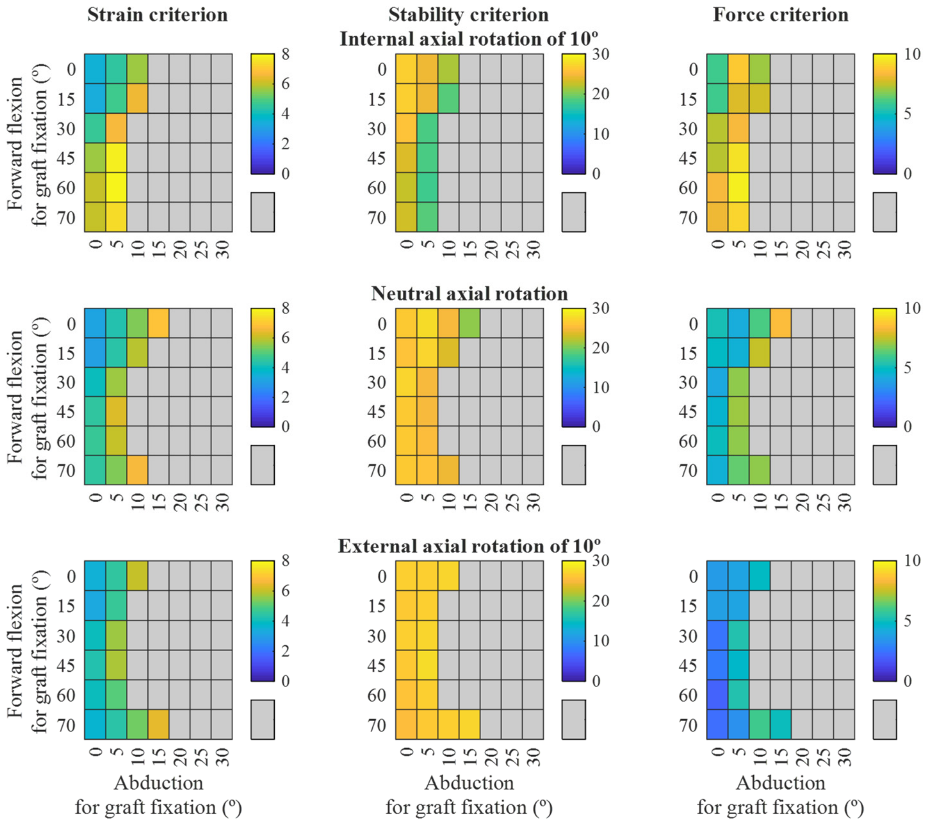

Figure S3. Heatmaps of the strain, stability, and force criteria for the shoulder condition (5), with implanted graft and a full-thickness tear of the supraspinatus and long head of the biceps tendons. The risk of failure increases as the strain criterion approaches 15%. Stability and force criteria values closer to 0 % means that the graft can better restore the stability of the shoulder joint to its healthy condition. The grey colour represents the shoulder positions of fixation that failed the screening step.
